# Disentangling the interactions between nasopharyngeal and gut microbiome and their involvement in the modulation of COVID-19 infection

**DOI:** 10.1128/spectrum.02194-23

**Published:** 2023-09-20

**Authors:** Leonardo Mancabelli, Giuseppe Taurino, Andrea Ticinesi, Tecla Ciociola, Federica Vacondio, Christian Milani, Federico Fontana, Gabriele Andrea Lugli, Chiara Tarracchini, Giulia Alessandri, Alice Viappiani, Massimiliano Bianchi, Antonio Nouvenne, Alfredo Antonio Chetta, Francesca Turroni, Tiziana Meschi, Marco Mor, Ovidio Bussolati, Marco Ventura

**Affiliations:** 1 Department of Medicine and Surgery, University of Parma, Parma, Italy; 2 Interdepartmental Research Centre "Microbiome Research Hub", University of Parma, Parma, Italy; 3 Department of Geriatric-Rehabilitation, Azienda Ospedaliero-Universitaria di Parma, Parma, Italy; 4 Department of Food and Drug, University of Parma, Parma, Italy; 5 Laboratory of Probiogenomics, Department of Chemistry, Life Sciences and Environmental Sustainability, University of Parma, Parma, Italy; 6 GenProbio srl, Parma, Italy; Università Roma Tre, Rome, Italy

**Keywords:** COVID-19, nasopharyngeal microbiota, gut microbiome, microbiome, inflammation

## Abstract

The human organism is inhabited by trillions of microorganisms, known as microbiota, which are considered to exploit a pivotal role in the regulation of host health and immunity. Recent investigations have suggested a relationship between the composition of the human microbiota and COVID-19 infection, highlighting a possible role of bacterial communities in the modulation of the disease severity. In this study, we performed a shotgun metagenomics analysis to explore and compare the nasopharyngeal microbiota of 38 hospitalized Italian patients with and without COVID-19 infection during the third and fourth pandemic waves. In detail, the metagenomic analysis combined with specific correlation analyses suggested a positive association of several microbial species, such as *S. parasanguinis* and *P. melaninogenica*, with the severity of COVID-19 infection. Furthermore, the comparison of the microbiota composition between the nasopharyngeal and their respective fecal samples highlighted an association between these different compartments represented by a sharing of several bacterial species. Additionally, lipidomic and deep-shotgun functional analyses of the fecal samples suggested a metabolic impact of the microbiome on the host’s immune response, indicating the presence of key metabolic compounds in COVID-19 patients, such as lipid oxidation end products, potentially related to the inflammatory state. Conversely, the patients without COVID-19 displayed enzymatic patterns associated with the biosynthesis and degradation of specific compounds like lysine (synthesis) and phenylalanine (degradation) that could positively impact disease severity and contribute to modulating COVID-19 infection.

**IMPORTANCE:**

The human microbiota is reported to play a major role in the regulation of host health and immunity, suggesting a possible impact on the severity of COVID-19 disease. This preliminary study investigated the possible correlation between nasopharyngeal microbiota and COVID-19 infection. In detail, the analysis of the nasopharyngeal microbiota of hospitalized Italian patients with and without COVID-19 infection suggested a positive association of several microbial species with the severity of the disease and highlighted a sharing of several bacteria species with the respective fecal samples. Moreover, the metabolic analyses suggested a possible impact of the microbiome on the host's immune response and the disease severity.

## INTRODUCTION

COVID-19 is a respiratory disease caused by the coronavirus SARS-CoV-2, involving more than 750 million cases worldwide from more than 200 countries (https://covid19.who.int/). Although COVID-19-related symptoms are mainly inherent to the respiratory system, recent studies have suggested a possible relationship between gut microbiota and disease onset, development, and severity (1 – 4).

The gut microbiota is defined as the community of microorganisms that live in the gastrointestinal tract. These microorganisms are reported to play a crucial role in digestive health, immune function, and other physiological processes (5, 6). In this context, several studies have suggested that alterations in the gut microbiota composition may be associated with increased susceptibility to viral infections (7, 8) and disease severity (1, 2). Indeed, comparative metagenomic analyses of the gut microbiota of healthy individuals and COVID-19 patients have reported differences in bacterial richness and composition (1, 2), revealing that COVID-19 patients were mainly characterized by a low bacterial richness and by reduced representation of beneficial bacteria, such as *Faecalibacterium*, *Roseburia*, and *Blautia*, as opposed to the increase of several opportunistic pathogen bacteria, such as *Actinomyces*, *Rothia*, and *Streptococcus* (9, 10). Moreover, the gut microbiota seems to be related to plasma concentrations of several cytokines, chemokines, and inflammation markers, suggesting its possible role in the modulation of host immune responses (2).

Similarly, the nasopharyngeal microbiota, i.e., the community of microorganisms living in the nasal cavities and throat, may also play a role in COVID-19 infections, since it is known that its changes could affect the severity and duration of respiratory diseases (11 – 13). In particular, several studies reported a decrease in the biodiversity (14) and significant differences in bacterial composition (13, 15) of the nasopharyngeal microbiota of SARS-CoV-2-infected patients compared to healthy controls. However, results obtained from different studies on the associations between the nasopharyngeal microbiota and COVID-19 disease provided contradictory data (12), revealing a huge level of variability. The contradictory results could also be related to the low taxonomic resolution analysis methodology used, mainly 16S rRNA gene microbial profiling, and the small sample size (12).

This study allowed the microbial profiling of the nasopharyngeal and gut microbiota of COVID-19 patients at a high taxonomic resolution and detected shared bacterial taxa in the human gut and nasopharyngeal districts, thus suggesting the occurrence of a clear bacterial link between these different body-compartments. Furthermore, specific lipidomic assays of fecal samples identified an intriguing relationship between microbial-driven metabolic pathways and the severity of COVID-19 infection.

## RESULTS AND DISCUSSION

### Clinical features and inflammatory profile of enrolled patients

A total of 38 Italian hospitalized patients (19 with COVID-19 and 19 who tested negative for SARS-CoV-2) were included in this study, and an overview of their clinical characteristics is provided in Table 1 and Table S1. COVID-19-negative patients were hospitalized mainly for cardiovascular and respiratory issues unrelated to COVID-19 infection (Table 1 and Table S1). Patients with positive RT-PCR for SARS-CoV-2 were classified as having severe COVID-19 in 14 cases and mild/moderate disease in 5 cases (Table S1).

**TABLE 1:** Clinical-laboratory characteristics of the studied population (*n* = 38)^*a*^.

	COVID negative N.19	COVID positive N.19	*P* value
Age (years)	79 (64–94)	56 (31–94)	**<0.001**
Female (%)	53	42	0.529
Fever (%)	21	79	**<0.001**
Cough (%)	5	58	**<0.001**
Subjective dyspnea (%)	26	32	0.729
Hypertension (%)	79	26	**<0.001**
Cardiopathy (%)	37	11	0.059
Dyslipidemia (%)	37	11	0.059
COPD (%)	37	5	**0.016**
Diabetes (%)	37	5	**0.016**
CKD (%)	11	0	0.154
Cerebrovascular disease (%)	16	0	0.074
Peripheral vascular disease (%)	21	0	**0.035**
Cognitive impairment (%)	5	0	0.324
Visual scoring of chest CT (%)	-	20 (10–35)	-
CRP, mg/L	34 (6–93)	59 (23–101)	0.198
PCT, ng/mL	0.17 (0.06–0.27)	0.10 (0.05–0.27)	0.563
IL-6, pg/mL	24 (13–134)	11 (5–20)	**0.010**
IL-10, pg/mL	1.7 (0.1–6.7)	8.4 (1.7–16.9)	0.150
Hospitalization duration (days)	8 (5–11)	11 (7–16)	0.146

^*a*^ Data are expressed as median and interquartile range or as percentages. The *P* value was calculated by Mann-Whitney or chi-square test. Values of *P* value < 0.05 are shown in bold. CKD, chronic kidney disease; COPD, chronic obstructive pulmonary disease; CRP, C-reactive protein; IL-6, interleukin 6; IL-10, interleukin 10; PCT, procalcitonin.

The inflammatory profiles of the enrolled subjects, based on C-reactive protein (CRP), procalcitonin (PCT), interleukin-6 (IL-6), and interleukin-10 (IL-10) assays, were assessed through the quantification of each marker in blood samples (Table 1). Overall, no significant differences between COVID-positive and COVID-negative patients were observed, except for pro-inflammatory cytokine IL-6, whose levels were lower in COVID-positive patients (*P* value = 0.01, Mann-Whitney test). Several factors may explain this result, such as age or the presence of other diseases in hospitalized COVID-negative subjects. Conversely, the anti-inflammatory cytokine IL-10 exhibited a trend toward higher in COVID-positive patients (Table 1). IL-6 and IL-10 showed a positive correlation only in COVID-positive patients (Spearman *r* = 0.42, *P* value one-tail = 0.036), suggesting a feedback mechanism as already proposed in the literature (16) (Fig. S1a).

### Intra- and inter-individual variability of the microbiota composition among nasopharyngeal samples of COVID-19-negative and COVID-19-positive patients

In order to identify possible differences in nasopharyngeal microbiota composition between patients with and without COVID-19 disease, 38 nasopharyngeal swabs were collected from all subjects, immediately inactivated with DNA/RNA shield buffer (Zymo Research, USA) and submitted to bacterial DNA extraction (17). Then samples were analyzed through the shallow shotgun metagenome sequencing approach, and the achieved data were processed and analyzed using the METAnnotatorX2 bioinformatics platform (18, 19), following the standard filtering parameters reported in the manual as previously reported (1). In detail, the bioinformatic analyses resulted in 1,385,606 reads with an average per sample of 36,463 ± 28,943 after quality and human sequences filtering (Table S1).

**Fig 1:**
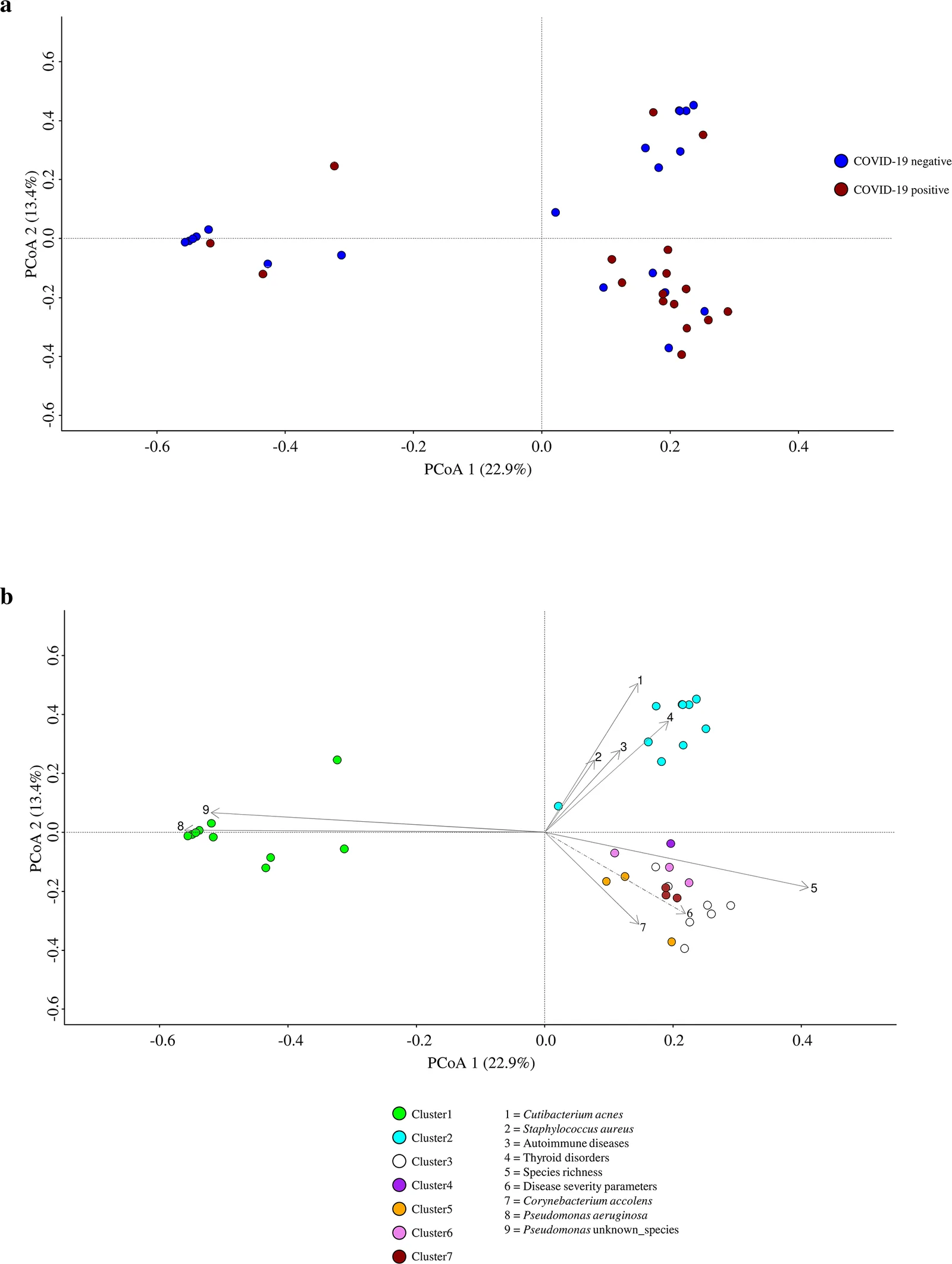
Evaluation of microbial biodiversity. Panel (a) shows the nasopharyngeal samples' principal coordinate analysis (PCoA), subdivided by COVID-19 disease. Panel (b) reveals the nasopharyngeal samples' PCoA, subdivided by the cluster identified through the unsupervised Elbow and Hierarchical CLustering analysis.

The results obtained by METAnnotatorX2 software were used to evaluate the microbial biodiversity of each nasopharyngeal sample. In detail, a beta-analysis represented through a tridimensional principal coordinates analysis (PCoA) based on the Bray-Curtis dissimilarity matrix revealed the absence of specific cluster subdivision based on the positivity/negativity of disease (PERMANOVA *P* value > 0.05), indicating possible independence of the nasopharyngeal microbiota composition from the pathological condition of the patients (Fig. 1a).

### Species-level taxonomic profiling of the nasopharyngeal microbiota

Detailed species-level taxonomic profiling of the 38 nasopharyngeal swabs was established through the METAnnotatorX2 software and used to identify possible microbial clusters based on taxonomic profiling patterns. An unsupervised Elbow approach was used to predict the number of clusters, revealing an optimal number of seven clusters (Fig. S1b). Therefore, a Hierarchical CLustering (HCL) analysis was used to classify the samples into the seven predicted clusters, also supported and represented by PCoA based on the Bray-Curtis dissimilarity matrix (Fig. 1b). The number of samples in each cluster ranged from 1 to 11 for Cluster 4 and Cluster 1, respectively, indicating variability in the composition of the nasopharyngeal microbiota. However, the less representative clusters identified in this preliminary analysis will need to be confirmed in more extensive and more heterogeneous populations from different world nations. Moreover, the PCoA based on the Bray-Curtis dissimilarity index was used to fit the main parameters of patients (Table S1), such as species richness, gender, age, vaccination, interleukin levels, patients' pathologies, and disease severity, i.e., negative, moderate, and severe, with the microbial species that composed the nasopharyngeal microbiota. The PCoA analysis revealed a possible correlation between several species and specific clusters (Fig. 1b). In detail, Cluster 1 showed a positive relationship with the *Pseudomonas aeruginosa* species (envit fit *P* value = 0.034, *r*² =0.960), Cluster 2 highlighted a positive correlation with *Cutibacterium acnes* (envit fit *P* value = 0.034, *r*² =0.851) and *Staphylococcus aureus* species (envit fit *P* value = 0.034, *r*² =0.203), while the remaining clusters, i.e., Clusters 3, 4, 5, 6, and 7, revealed a possible association with *Corynebacterium accolens* species (envit fit *P* value = 0.034, *r*² =0.363).

Furthermore, the fits analysis revealed a negative association of species richness (envit fit *P* value = 0.005, *r*² =0.286) with Clusters 1 and 2, while Clusters 3, 4, 5, 6, and 7 resulted in a positive association (Fig. 1b). Moreover, parameters regarding autoimmune diseases and thyroid disorders display a positive trend toward Cluster 2, suggesting a possible relationship between *C. acnes* and *S. aureus* species and these conditions. In addition, the disease severity parameters did not show a significant fit (envit fit *P* value = 0.157, *r*² =0.105), but suggested a trend related to the increase in species richness (Fig. 1b), pointing out a possible correlation between the complexity of the nasopharyngeal microbiota and the severity of COVID-19 clinical outcomes.

To better investigate the possible correlation between disease severity and the nasopharyngeal microbiota composition, a specific Spearman’s correlation analysis was performed. In detail, the correlation analysis included the values of IL-6 and IL-10, the predicted species composition, the disease severity, and the species richness value of each sample (Table S2). Therefore, the analysis suggested a possible negative correlation between *P. aeruginosa* and the severity of disease (*R* = −0.472, *P* value = 0.027), as well as *Enterocloster bolteae* (*R* = −0.458, *P* value = 0.036), while *Streptococcus parasanguinis* (*R* = 0.451, *P* value = 0.041), *Streptococcus* unknown species (*R* = 0. 549, *P* value = 0.005), *Prevotella melaninogenica* (*R* = 0.451, *P* value = 0.041), *Actinomyces* unknown species (*R* = 0.501, *P* value = 0.015), and *Schaalia* unknown species (*R* = 0.501, *P* value = 0.015) resulted positively correlated with increasing disease severity. Moreover, a specific multivariate analysis through MaAslin2 software (20) and based on the values of IL-6 and IL-10, the predicted species composition, the disease severity, and the species richness confirmed the possible negative correlation between the *E. bolteae* (*R* = −0.834, *P* value = 0.034) but also suggested the negative correlation with *Faecalibacterium prausnitzii* (*R* = −0.848, *P* value = 0.046) (Table S2). Furthermore, the multivariate analysis confirms the positive correlation between the increasing gravity of the disease and *Streptococcus* unknown species (*R* = 0.861, *P* value = 0.042) and *Actinomyces* unknown species (*R* = 0.347, *P* value = 0.046) (Table S2). Such data mostly confirmed previously published data about the COVID-19 nasopharyngeal microbiota composition, confirming the possible correlation between species belonging to *Prevotella* and *Streptococcus* and disease severity (14, 15, 21 – 25) and reinforcing the notion of the existence of microbial markers associated with the gravity of symptoms following COVID-19 infection (26).

### Identification of shared microbial taxa between nasopharyngeal and fecal microbiota

Several studies suggested possible bacterial sharing between the oral cavity and gastrointestinal tract in human beings, defining this as the oral-gut microbiota axis (27 – 29). Moreover, the alteration of the oral-gut microbiota axis eubiosis, i.e., the modification of the bacterial composition, has been associated with several human diseases and disorders, such as colitis, inflammatory bowel disease (IBD), and neurodegenerative diseases (27 – 29). In this context, the deep-shotgun metagenomics approach of the fecal samples (Table S3) was used to reconstruct the metagenome-assembled genomes (MAGs) and applied to the identification of microbial taxa shared between the nasopharyngeal and the gut microbiotas using a strain-tracking approach (30). Therefore, short reads obtained from shallow-shotgun metagenomics data from the patients' nasopharyngeal swabs were aligned on the MAGs obtained from the respective fecal samples. In detail, 66% of the nasopharyngeal samples showed at least one match with at least one fecal reference MAGs (Table 2). This preliminary analysis of the shared bacterial species revealed that 11 species are exclusively shared between nasopharyngeal and fecal samples of COVID-19 patients, while 16 species were solely shared between COVID-19-negative samples (Table 2). Interestingly, the microbial species shared by the samples with COVID-19 include species belonging to the *Bacteroides* genus, such as *Bacteroides ovatus*, *Bacteroides xylanisolvens*, which have already been observed to be positively correlated to increased disease severity (31, 32). In contrast, COVID-19-negative samples showed the sharing of potentially beneficial bacterial species, such as *F. prausnitzii*, that is hypothesized to be involved in producing several anti‐inflammatory compounds (2, 32). These preliminary results confirm the possible relationship in the microbial community composition between different human body compartments, e.g., nasopharynx and gut, suggesting a bidirectional interaction that could be involved in the host’s health or pathologic immune responses.

**TABLE 2:** Shared microbial taxa between nasopharyngeal and fecal microbiota^*a*^

	Neg2	Neg4	Neg6	Neg9	Neg11	Neg12	Neg13	Neg14	Neg15	Neg16	Neg17	Neg18	Neg19	Mod1	Mod2	Mod4	Sev1	Sev2	Sev3	Sev4	Sev5	Sev11	Sev12	Sev13	Sev14
*Akkermansia* unknown species												**▲**													
*Alistipes communis*							**▲**	**▲**																	
*Alistipes onderdonkii*							**X**													**X**					
*Alistipes putredinis*													**X**				**X**								
*Alistipes* unknown species																							●		
*Bacteroides caccae*																								●	
*Bacteroides eggerthii*						**▲**																			
*Bacteroides ovatus*																						●			●
*Bacteroides stercoris*												**X**												**X**	**X**
*Bacteroides thetaiotaomicron*										**▲**															
*Bacteroides togonis*													**▲**												
*Bacteroides uniformis*								**X**							**X**				**X**					**X**	**X**
*Bacteroides xylanisolvens*																●								●	
*Barnesiella intestinihominis*				**▲**																					
*Collinsella tanakaei*	**▲**																								
*Duodenibacillus* unknown species													**▲**												
*Enterocloster bolteae*																●									
*Eubacterium rectale*											**▲**														
*Faecalibacterium prausnitzii*												**▲**	**▲**												
*Klebsiella aerogenes*			**▲**										**▲**												
*Klebsiella pneumoniae*						**▲**																			
*Mesosutterella multiformis*													**▲**												
*Parabacteroides distasonis*																		●							
*Parabacteroides merdae*					**▲**																				
*Phocaeicola dorei*																●								●	
*Phocaeicola massiliensis*																●									
*Phocaeicola plebeius*																		●							
*Phocaeicola vulgatus*														●		●					●	●			
*Prevotella copri*								**X**					**X**					**X**							
*Prevotella pectinovora*													**▲**												
*Prevotella* unknown species									**X**														**X**		
*Roseburia inulinivorans*		**▲**																							
*Ruminococcus* unknown species														●											

^*a*^ The circles highlighted the bacteria exclusively shared between nasopharyngeal and fecal samples of patients with COVID-19, while triangles highlighted the species solely shared in COVID-19-negative samples.

### Lipidomic analyses of fecal samples

**Fig 2:**
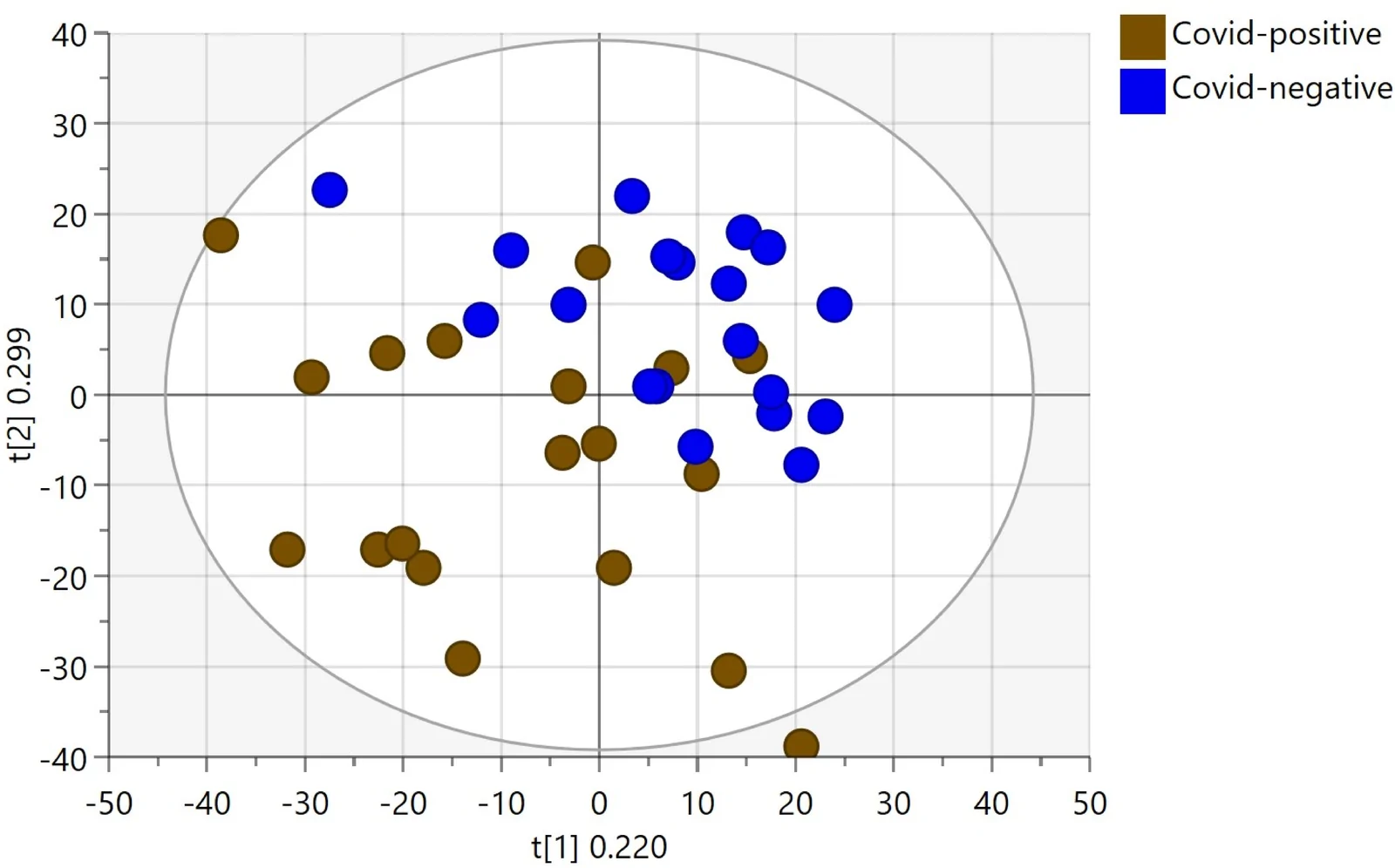
Lipidomic profiles of fecal samples. Two-factor PLS-DA score plot based on all UHPLC-HRMS features (*n* = 2,031) on COVID-19-positive (brown) and COVID-19-negative (blue) groups.

The UPLC-HRMS features (*n* = 2,031) extracted from the analytical batch underwent partial least squares discriminant analysis (PLS-DA). The two-factor PLS-DA score plot (*R* ^2^_*X*_ = 0.45, *R* ^2^_*Y*_ = 0.52 Fig. 2) showed that a combination of *t* (1) and *t* (2) was able to define a separation between the COVID-19-positive (*n* = 19) and COVID-19-negative (*n* = 19) cohorts, albeit with some exceptions (three COVID-positive samples within the COVID-negative group) (Fig. 2). Through comparison of HRMS features (accurate mass, fragmentation spectra, and isotopic similarity) with database references (Lipid Maps and HMDB), a putative identity was assigned to 321 signals over 2,031 detected. As shown in Table 3, among the most discriminant features of PLS-DA model (VIP > 1.7), two fatty aldehydes (FAL 19:0; FAL 19:1), a fatty alcohol (FOH 17:2) and one phosphatidylethanolamine (PE 34:1) were identified, whose intensities were significantly higher (*P* < 0.01 at unpaired two-tailed *t* test) in COVID-positive than in COVID-negative group (Tables S4 and S5; Fig. S1c). Medium- to long-chain aliphatic aldehydes and alcohols do not represent essential components of cellular membranes, and they do not accumulate to a significant extent as free lipids; they are, instead, either used as substrates for the biosynthesis of other lipids or as catabolic intermediates that are subsequently metabolized (33). Oxidative stress produces a wide variety of short- and medium-chain aldehydes that originate from peroxidation of polyunsaturated fatty acids by reactive oxygen species (ROS), such as malonaldehyde (MDA), or 4-hydroxy-2-alkenales (34, 35). Long-chain aliphatic aldehydes are largely produced by catabolic metabolism of ether glycerolipids, fatty alcohols, sphingolipids, and wax esters; e.g., ROS were shown to oxidatively attack the 1-O-alkenyl vinyl ether bond of plasmalogen lipids, rich in polyunsaturated fatty acids (PUFA) to release the alkyl chain as fatty aldehydes (36). When they accumulate beyond physiological levels, fatty aldehydes are toxic to cells due to their propensity to form covalent adducts with other molecules, e.g., long-chain aldehydes are known to form Schiff base adducts with free amino groups in proteins and lipids (37). The increase of FAL-related signals in the COVID-positive cohort can therefore be related to the oxidative stress resulting from COVID-19 infection in the gut (25, 38). A representative of the PE class (PE 34:1) was also positively modulated in the COVID-positive cohort. Lipid reprogramming toward the rise in systemic PE has been recently described as a prognostic feature of critical illness resulting from different etiologies (39). The authors proposed a lipid reprogramming score derived from PE as a risk factor and correlated it with excessive pro-inflammatory response and worse outcomes in two trauma and two COVID-19 data sets (39 – 41). Since one of the key organs for systemic lipogenesis and lipolysis is the liver, the increase in the fecal content of PE and TG within the COVID-positive cohort could indicate a condition of hyper-metabolism associated with a critical condition of systemic inflammation and metabolic stress resulting from COVID-19 infection (39).

**TABLE 3:** Lipid metabolites filtered according to the PLS-DA model (VIP values > 1.7) and showing significantly different average intensities in COVID-positive and COVID-negative cohorts (*P* < 0.01)

Putative identity^*a*^	Lipid class	VIP^*b*^	COVID-negative^*c*^	COVID-positive^*c*^	*P* value^*d*^
FAL 19:0	Fatty aldehyde	2.39	−0.49 ± 0.31	1.37 ± 0.35	0.0003
FOH 17:2	Fatty alcohol	1.93	1.93 ± 0.14	2.65 ± 0.15	0.001
FAL 19:1	Fatty aldehyde	1.73	1.48 ± 0.13	2.15 ± 0.17	0.004
PE 34:1	Diacylphosphatidylethanolamine	1.89	0.91 ± 0.31	2.12 ± 0.25	0.005

^*a*^ FAL = Fatty Aldehyde; FOH = Fatty Alcohol; PE = Phosphatidylethanolamine.

^*b*^ Variable Importance in Projection (VIP) of the PLS-DA model > 1.7.

^*c*^ Log average intensity ± SEM (*n* = 19).

^*d*^ Significance was set at *P* < 0.01 by two-tailed unpaired *t* test.

### Correlation between microbiome and lipidomic profiles of fecal samples

The deep-shotgun metagenome sequencing of the fecal samples was used to predict the metabolic enzymatic reactions based on the MetaCyc database (42) and the Enzyme Commission (EC) classification. The enzymatic reactions identified were used to perform a Spearman’s correlation analysis, including the taxonomical profile at the species level, the lipidomic profile, and the main parameters of patients (Table S1), such as species richness, gender, age, vaccination, interleukin levels, patients' pathologies, status health, and disease severity. Focusing our interest on the results associated with the severity of COVID-19 infection (Table 4), the analysis corrected through the Benjamini-Hochberg procedure revealed a significant positive correlation with such patients' pathologies, i.e., cough, fever, and pneumonia, with species belonging to *Bacteroides* genus (*P* value = 0.017, correlation value 0.495) and with four lipid classes, including the compound FAL 19:0 and FOH 17:2, which have been already highlighted in patients with COVID-19 by lipidomic analysis (see above). Thus, altered gut microbiota, presenting an increased load of bacteria potentially involved in putrefactive dysbiosis (1, 43), and increased lipid compounds, such as aldehydes, representing metabolic products potentially related to the inflammatory state (38), could indicate a correlation of the human microbiome with the disease severity. Conversely, Spearman’s correlation analysis highlighted a negative correlation between the severity of the disease and 17 enzymatic reactions mainly related to biosynthesis and degradation of metabolites (Table 4). Interestingly, among these enzyme classes, 2.3.1.117 and 4.2.1.96 ECs were identified, related to lysine synthesis and phenylalanine degradation, respectively. These findings could suggest an interaction between the gut microbiota and the inflammatory condition of the host. In fact, recent studies reported that lysine and its derivative ester could play a role in inhibiting COVID-19 infection (44), while phenylalanine was reported to be associated with increased inflammation (45), thus suggesting the potential impact of the gut microbiome and its metabolic functionality on the inflammatory response of the host.

**TABLE 4:** Results of Spearman’s correlation analysis associated with the severity of COVID-19 disease^*a*^

	Spearman’s rank correlation coefficient	FDR *P* value (Benjamini and Hochberg correction)
1.1.1.14	−0.530003825	0.00776
1.1.1.264	−0.529173182	0.00791
1.2.1.70	−0.445304369	0.04164
1.2.4.2	−0.452368547	0.03688
2.3.1.117	−0.453896762	0.03590
2.7.1.22	−0.450305154	0.03823
2.7.7.56	−0.455135291	0.03514
2.7.8.37	−0.486374298	0.01964
3.5.1.125	−0.436541886	0.04817
3.5.3.11	−0.452683134	0.03668
3.5.4.28	−0.526251891	0.00845
4.1.1.-	−0.437027337	0.04781
4.2.1.41	−0.472808292	0.02550
4.2.1.96	−0.441295125	0.04455
4.3.1.15	−0.455353115	0.03500
5.1.3.29	−0.499165075	0.01519
5.3.1.22	−0.488254645	0.01893
Cer 32:1;O2	−0.450384877	0.03818
Cer 34:1;O2	−0.52829214	0.00806
Cer 36:0;O3	−0.44989179	0.03851
Cer 36:1;O2	−0.508388646	0.01251
16:0-Glc-Stigmasterol	−0.494540165	0.01670
LPE 18:1	−0.474423976	0.02474
Hypertension	−0.605042982	0.00109
Vaccination	−0.720946544	0.00001
3.2.1.78	0.477319776	0.02341
3.2.1.8	0.499650526	0.01503
3.4.14.2	0.487863752	0.01908
4.2.1.115	0.472829354	0.02550
10Z-Nonadecen-2-one	0.465426225	0.02924
FAL 19:0	0.544464387	0.00552
FOH 17:2	0.496616457	0.01600
PE 34:1	0.458872635	0.03289
*Bacteroides* unknown_species	0.495281467	0.01645
Cough	0.561164767	0.00365
Fever	0.715995512	0.00002
Pneumonia	0.727650811	0.00001

^*a*^ Only significant results were reported.

### Conclusion

An increasing number of studies have highlighted the occurrence of many correlations between human microbiota and human diseases and disorders. In this context, the possible associations between COVID-19 infection and human microbiota composition were suggested. Therefore, to explore the possible correlation between nasopharyngeal microbiota and COVID-19 infection, a preliminary shotgun metagenomic analysis was performed on nasopharyngeal swabs of 38 Italian hospitalized patients with and without COVID-19 during the third and fourth pandemic waves. This metagenomic analysis suggested that the severity of the disease could be linked to an increased complexity of bacterial communities and the occurrence of bacterial species belonging to the genera *Streptococcus*, *Prevotella*, *Actinomyces*, and *Schaalia*. Moreover, comparing the bacterial communities of each nasopharyngeal and fecal microbiota allowed the identification of shared bacteria between the two human compartments. Specifically, COVID-19-positive patients shared species belonging to the *Bacteroides* genus, while patients without COVID-19 shared potentially beneficial species, such as *F. prausnitzii*, suggesting a bidirectional interaction that could be involved in the immune responses of the host. Additionally, the correlation analysis between the main clinical parameters of patients as well as the lipidomic profile, and the metagenome sequencing of the fecal samples evidenced associations between the severity of COVID-19 infection and specific lipid and enzyme classes. Specifically, patients with COVID-19 showed a positive correlation with several lipidic compounds, such as aldehydes, while patients without COVID-19 revealed a positive correlation with enzyme classes related to lysine synthesis and phenylalanine degradation, suggesting the possible impact of the gut microbiome and its metabolic functionality on the inflammatory response of the host.

In conclusion, our study provides preliminary scientific evidence of a possible relationship between COVID-19 infection and the nasopharyngeal and fecal microbiota composition. However, our preliminary results need to be confirmed in larger, more heterogeneous populations from different world nations. Specifically, more detailed metadata from a larger cohort could allow the identification of possible confounding factors influencing the microbiome, such as age, diet, and sex, overcoming the inherent limitations of a preliminary pilot study. Moreover, future studies exploring the relationships between COVID-19 and the microbiota, as well as the integration of data from multiple sources, could provide a complete understanding of the complex bidirectional interplay between host and microbes. Thus, meta-analyses of similar studies could further advance our knowledge of the relationship between COVID-19, the nasopharyngeal and intestinal microbiota, and disease severity.

## MATERIALS AND METHODS

### Patient enrollment and data collection

A group of patients hospitalized with reverse transcriptase-polymerase chain reaction (RT-PCR) nasopharyngeal swabs positive for SARS-CoV-2 was enrolled in the COVID-19 Internal Medicine ward of Parma University-Hospital in Italy during the third and fourth pandemic waves. Included were patients over 18 years old who tested positive for SARS-CoV-2 no more than 72 h before enrollment, irrespective of symptoms and pulmonary involvement on chest imaging. Patients with respiratory symptoms (cough, dyspnea, and fever), peripheral oxygen saturation <94% in room air, and presence of at least 5% of pulmonary involvement of lung parenchyma (consolidations and ground-glass abnormalities) on chest computed tomography (CT) were classified as severe COVID-19 cases. Conversely, subjects with oxygen saturation in room air >94% and only minor or absent involvement of lung parenchyma on chest CT were classified as mild/moderate COVID-19 cases.

A group of patients hospitalized in an Internal Medicine ward of Parma University-Hospital in the same period for reasons other than COVID-19 was also enrolled as control group. Patients included in this group were older than 18 years old, tested negative on RT-PCR for SARS-CoV-2 performed upon ward admission, and had oxygen saturation >94% in room air without need of oxygen support.

Excluded from both groups were patients with dementia or severe cognitive impairment, active malignancy, severe respiratory failure needing immediate ventilatory support, severe immunological disorders, active gastrointestinal diseases associated with gut microbiota dysbiosis, and patients who had undergone systemic antibiotic treatment for more than 5 days upon first evaluation.

After signing an informed consent form, all study participants provided two fecal samples for microbiome and lipidomic analyses. A nasopharyngeal swab was also collected for microbiome analyses, alongside with a blood sample for cytokine profiling.

Clinical metadata was also collected from each patient’s records, including age, gender, comorbidities, chest CT findings, symptoms, level of oxygen support administered, arterial blood gas analysis findings, and levels of routine blood tests, such as CRP and PCT.

For cytokine determination, samples were centrifuged at 3,500 rpm for 10 min to separate cells from serum, which was then used to quantify IL-6 and IL-10 with specific human ELISA kits (Demeditec Diagnostic GmbH, Germany), following the manufacturer’s instructions.

The study protocol was approved by the local ethics committee (Comitato Etico dell'Area Vasta Emilia Nord, Emilia-Romagna Region, Italy), under the ID 1131/2020/TESS/UNIPR. All procedures were performed in compliance with the Declaration of Helsinki principles.

### Collection of nasopharyngeal and fecal samples

The nasopharyngeal swabs obtained from the patients were immediately inactivated with DNA/RNA shield buffer (Zymo Research) and subsequently submitted to bacterial DNA extraction using the previously described protocol (17). Similarly, approximately three grams of fresh stool samples were collected from each Italian hospitalized patient using a dedicated sterile tube containing the DNA/RNA shield buffer (Zymo Research). After collection, stool samples were immediately shipped to the laboratory and further processed as previously described (1).

### Shallow- and deep-shotgun sequencing

According to the manufacturer’s instructions, DNA library preparation was performed using the Nextera XT DNA sample preparation kit (Illumina, San Diego, CA, USA). First, 1 ng input DNA from each sample was used for the library preparation, which underwent fragmentation, adapter ligation, and amplification. Then, Illumina libraries were pooled equimolarly, denatured, and diluted to a concentration of 1.5 pM. Next, DNA sequencing was performed on a MiSeq instrument (Illumina) using a 2 × 250 bp output sequencing kit together with a deliberate spike-in of 1% PhiX control library.

### Taxonomic classification of sequence reads

Taxonomic profiling of sequenced reads was performed by employing the METAnnotatorX2 bioinformatics platform (18, 19). In detail, the fastq files obtained from sequencing were filtered to remove reads with quality of <25, and to retain reads with a length of >100 bp. Subsequently, a human host DNA filtering was performed through bowtie2 software (46, 47), following the METAnnotatorX2 manual (19). Afterward, the taxonomic classification was achieved by means of MegaBLAST (48) employing a manually curated and pre-processed database of genomes retrieved from the National Center for Biotechnology Information, following the METAnnotatorX2 manual (19).

### Functional prediction

Functional profiling of the sequenced reads was performed with the METAnnotatorX2 bioinformatics platform (18, 19). Functional classification of reads was performed to reveal metabolic pathways based on the MetaCyc database (release 24.1) (42) through RAPSearch2 software (49, 50).

### Lipidomic analyses of fecal samples

#### Chemicals and reagents

Ultrapure water was prepared by a Milli-Q plus system from Millipore (Bedford, MA, USA). LC-MS grade acetonitrile (MeCN), methanol (MeOH), isopropanol (IPA), acetic acid, and ammonium acetate were supplied by Scharlab (Barcelona, Spain). Methyl tert-butyl ether (MTBE) was purchased from Sigma-Aldrich (Milan, Italy). Major Mix IMS/TOF calibration kit and Leukin-Enkephalin (Leu-Enk) solution for external and internal calibration of the high-resolution mass spectrometer were purchased from Waters (Manchester, UK).

#### Sample preparation

Fecal samples (*n* = 19 COVID-19-positive and *n* = 19 COVID-19-negative patients) were processed employing a published liquid-liquid extraction protocol for the isolation of the lipidome, with minor modifications (51). Briefly, fecal slurry (200 µL) was extracted with a mixture of MeOH:MTBE (3:10 vol/vol) and, after 1 h of incubation on shaker, ultrapure water (1.25 mL) was added to induce phase separation. After further 10 min of incubation on shaker, samples were centrifuged (5,000 × *g*, 5 min, 4°C) and 1 mL of the upper organic phase was dried under a gentle nitrogen stream. Dried lipid extracts were dissolved in 200 µL MeCN:IPA:water (1:2:1, vol/vol/vol) immediately before Ultra Performance Liquid Chromatography-High Resolution Mass Spectrometry (UPLC-HRMS) analysis.

#### UPLC-HRMS lipidomic analysis

An Acquity UPLC system (Waters, Milford, MA, USA) interfaced with a VION ion mobility hybrid Quadrupole-Time of Flight Mass Spectrometer (IMS-Q-ToF MS) (Waters) was used to analyze fecal metabolites. Extracts were injected into an Acquity UPLC-HSS T3 column (2.1 × 100 mm^2^, 1.7 µm; Waters) thermostated at 55°C. Samples were analyzed by gradient elution employing as solvent A: MeCN:water (40:60 vol/vol) and as solvent B: IPA:water:MeCN (90:5:5 vol/vol/vol) both containing 5 mM ammonium acetate and 0.1% vol/vol acetic acid. Gradient was as follows: *t*(0 min): 60%A:40%B; *t*(14 min): 100%B; *t*(17 min): 100%B; and *t*(20 min): 60%A:40%B. at a flow rate of 0.4 mL/min. Metabolites separated by UPLC were analyzed in ESI-positive mode. The capillary and sampling cone voltages were set at 2.50 kV and 40 V, respectively. The desolvation flow was set to 1,000 L/h at a temperature of 500°C and the source temperature was set to 120°C. The IMS-Q-ToF MS data were collected in the range of mass-over-charge ratio (*m/z*) 50–1,200 with a scan time of 0.2 s and interscan delay time of 0.02 s. External calibration of mass and drift time values was carried out before the analysis of the analytical batch employing the MajorMix IMS/TOF calibration kit prepared following the manufacturer’s instructions at a flow rate of 20 µL/min. Internal lock mass calibration was used to ensure accuracy in mass measurement during the analyses. Leu-Enk (556.2771 Da in ESI-positive mode) was used as the lock mass at 50 ng/mL and at a flow rate of 10 µL/min, measured every 3 min. The software UNIFI v.1.8.2 (Waters) was used for system control and data acquisition. The acquisition method was data-independent high-definition MSE. A 5 eV energy scan allowed to acquire high-resolution mass values of precursor ions and a ramped high energy scan (20–40 eV) induced a simultaneous fragmentation of the same ions. The analytical batch was constituted of randomized blank samples, QC samples, and unknown samples. QC samples were prepared by mixing 10 µL aliquots of every sample in analysis. Ten QC sample injections were employed at the beginning of the analytical batch to ensure system stability and a QC sample was injected every 10 samples.

#### Data processing

Raw data were imported as a UNIFI .uep file into the untargeted analysis software Progenesis QI v.2.4 (Nonlinear Dynamics, UK), which performed automatic alignment of batch runs selecting the most suitable QC sample in the batch as alignment reference. Data filtering was carried out with the following cutoffs: (i) observed variance in the QC samples, expressed as percent coefficient of variation (CV) <25%; (ii) *m/z* ≥250; and (iii) at least 60% of values over samples different from 0. A total of 2,031 ion features were retrieved. Metabolite assignment was performed by elemental composition analysis with calculated mass, mass tolerance (ppm), isotopic similarity (calculated isotopic pattern vs experimental one), and mass fragmentation by searching into ChemSpider (www.chemspider.com) and Lipid Maps Database (https://www.lipidmaps.org/).

### Statistical analysis

ORIGIN 2021 (https://www.originlab.com/2021) was used to compute statistical analyses, including HCL and Silhouette analyses. Moreover, the similarities between samples (beta-diversity) were calculated by the Bray-Curtis dissimilarity matrix based on species abundance, using the “vegdist” function (from vegan_2.5-7) on RStudio (http://www.rstudio.com/). The range of similarities is calculated between values 0 and 1. Beta-diversity was represented through PCoA using the function “ape” of the R suite package (52). Moreover, the available metadata and the various detected bacterial species were tested and plotted on the PCoA using the “envfit” and “plot” functions from vegan (version 2.5-7), respectively, through RStudios (http://www.rstudio.com/). PERMANOVA analyses were performed on RStudio using 999 permutations to estimate *P* values for population differences in PCoA analyses with adonis2 package (from vegan_2.5-7). The false discovery rate (FDR) correction is applicated to all statistical analyses based on Benjamini and Hochberg correction (53), using RStudio through “p.adjust” function (from base package stats). Furthermore, a correlation analysis between the available metadata and the various detected bacterial species of all samples was performed through Spearman’s rank correlation coefficient using “rcorr” function (from Hmisc_4.6-0, https://CRAN.R-project.org/package=Hmisc), and only significative statistical results were retained. Moreover, the FDR correction based on Benjamini and Hochberg correction (53) and calculated using RStudio through “p.adjust” function (from base package stats) was applied to statistically significant results. Moreover, multivariate analyses were performed through MaAsLin2 software (20), reporting the *P* value corrected through Benjamini and Hochberg correction. The UPLC-HRMS lipidomic data set was analyzed by the multivariate statistical analysis package SIMCA-P+ version 12.0.2 (Umetrics, Umeå, Sweden). PLS-DA was used to visually discriminate between COVID-19-positive and COVID-19-negative groups. To find metabolites that contributed to the discrimination between the two groups, log differences of the metabolite intensities in COVID-positive and COVID-negative groups were tested by two-tailed unpaired *t* tests. Statistical significance was set at *P* < 0.01.

Statistical analysis of clinical data (Table 1) has been performed with Mann-Whitney or chi-square test.

## Data Availability

Raw sequences of the Italian shallow- and deep-shotgun metagenomics data are accessible, in anonymous form, through SRA under study accession number PRJNA975734.

## SUPPLEMENTAL MATERIAL

Figure S1 spectrum.02194-23-s0001.pdf Panel a reports the inflammatory profile of enrolled patients. Panel b shows the unsupervised Elbow calculated on the species-level taxonomic profiling of the 38 nasopharyngeal swabs collected. Panel c reports the Volcano plot on identified lipid metabolites (*n*=321). Y-axis: negative Logarithm of *P*-value in two-tailed unpaired *t*-test; X-axis: difference of mean Log intensities in the COVID-positive vs. COVID-negative cohorts. Significance was set at *P*<0.01 (-Log *P* > 2.00) and DeltaLogintensities < −0.5 and >0.5 (approx. 3-fold change).

Table S1 spectrum.02194-23-s0002.xlsx Metadata and filtering report of the nasopharyngeal samples sequenced in this study.

Table S2 spectrum.02194-23-s0003.xlsx Spearman's correlation and MaAslin2 analyses based on the values of IL-6 and IL-10, the predicted species composition, the disease severity, and the species richness value of each sample. Only significant results were reported.

Table S3 spectrum.02194-23-s0004.xlsx Filtering report of the fecal samples sequenced in this study.

Table S4 spectrum.02194-23-s0005.xlsx Lipid metabolites identified in COVID-positive and COVID-negative cohorts filtered according to the PLS-DA model (VIP values > 1.7).

Table S5 spectrum.02194-23-s0006.xlsx Lipid metabolites showing significantly different average intensities in COVID-positive vs. COVID-negative cohorts (*P*<0.01).

ASM does not own the copyrights to Supplemental Material that may be linked to, or accessed through, an article. The authors have granted ASM a non-exclusive, world-wide license to publish the Supplemental Material files. Please contact the corresponding author directly for reuse.
