# Supplementary material for: Disentangling the interactions between nasopharyngeal and gut microbiome and their involvement in the modulation of COVID-19 infection
Source: Microbiol Spectr. 2023 Sep 20;11(5):e02194-23. doi: 10.1128/spectrum.02194-23 (PMC10581039; doi:10.1128/spectrum.02194-23)
Supplement: Figure S1 — Panel a reports the inflammatory profile of enrolled patients. Panel b shows the unsupervised Elbow calculated on the species-level taxonomic profiling of the 38 nasopharyngeal swabs collected. Panel c reports the Volcano plot on identified lipid metabolites (n=321). Y-axis: negative Logarithm of P-value in two-tailed unpaired t-test; X-axis: difference of mean Log intensities in the COVID-positive vs. COVID-negative cohorts. Significance was set at P<0.01 (-Log P > 2.00) and DeltaLogintensities < −0.5 and >0.5 (approx. 3-fold change). [file spectrum.02194-23-s0001.pdf]

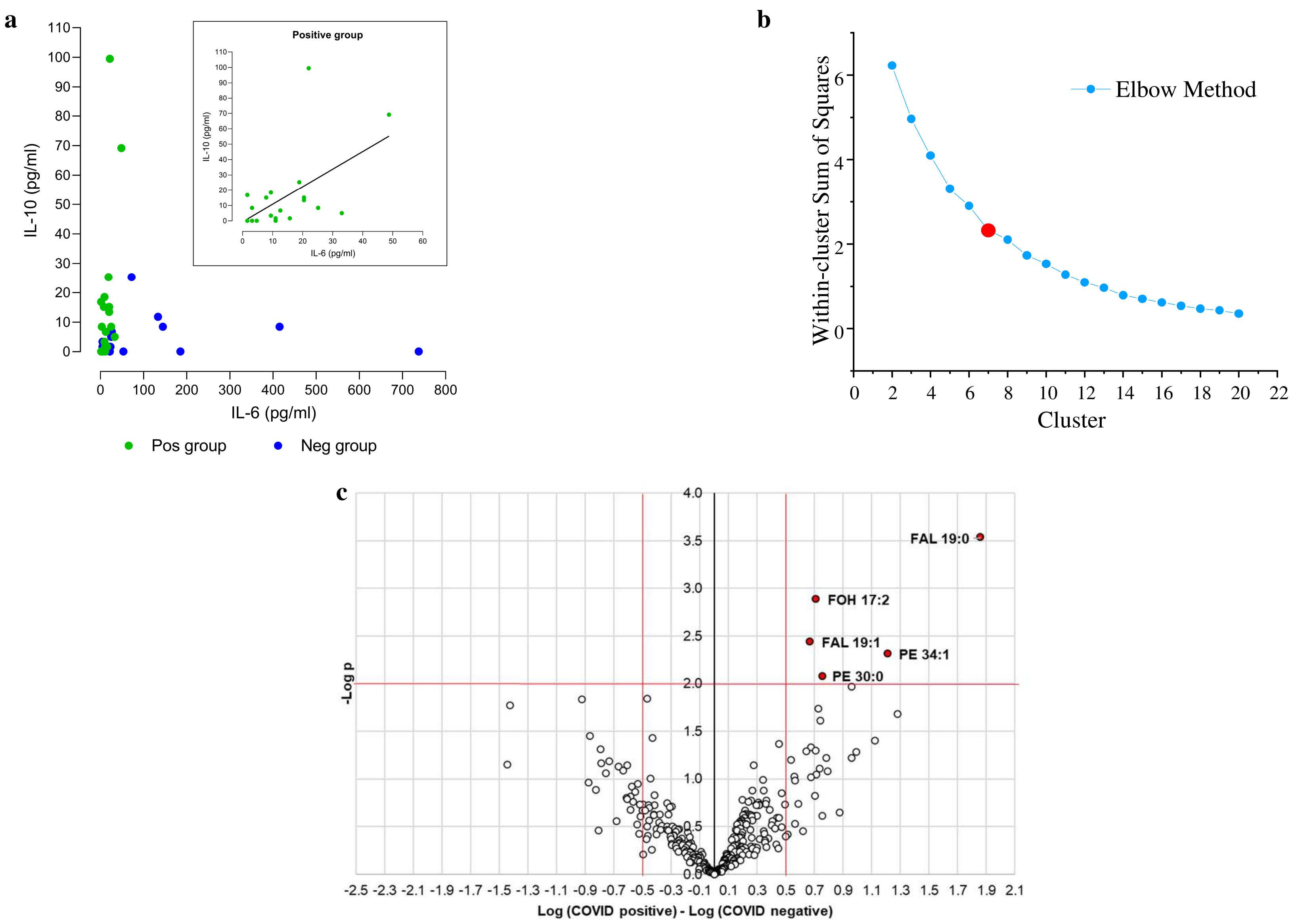

Figure S1. Panel a reports the inflammatory profile of enrolled patients.
Panel b shows the unsupervised Elbow calculated on the species-level taxonomic profiling of the 38 nasopharyngeal swabs collected.
Panel c reports the Volcano plot on identified lipid metabolites (n=321). Y-axis: negative Logarithm of p-value in two-tailed unpaired t-test; X-axis: difference of mean Log intensities in the COVID-positive vs. COVID-negative cohorts. Significance was set at $p<0.01$ (-Log p > 2.00) and DeltaLog intensities < -0.5 and >0.5 (approx. 3-fold change).
